# A Family-Focused Intervention for Parental Mental Illness: A Practitioner Perspective

**DOI:** 10.3389/fpsyt.2021.783161

**Published:** 2021-11-23

**Authors:** Mairead Furlong, Christine Mulligan, Sharon McGarr, Siobhan O'Connor, Sinead McGilloway

**Affiliations:** Centre for Mental Health and Community Research, Maynooth University, Maynooth, Ireland

**Keywords:** family talk, implementation, mental disorder, mental illness, parents, qualitative, COPMI, children

## Abstract

**Background:** Parental mental illness (PMI) is common and can lead to children developing mental disorders. Family Talk (FT) is a well-known and widely implemented intervention designed to reduce the risk of transgenerational psychopathology. However, given the research to practise “gap,” very little qualitative research, to date, has investigated practitioner experiences in implementing FT. This study aimed to explore the practitioner-perceived barriers and facilitators to the implementation and sustainability of FT within mainstream mental health settings.

**Methods:** This qualitative study was nested within a randomised controlled trial (RCT) of Family Talk [*N* = 86 families (139 parents, 221 children)] within 15 adult (AMHS), child (CAMHS), primary care mental health, and child protection sites in Ireland. Semi-structured interviews and focus groups were undertaken with a purposive sample of clinicians (*n* = 31) and managers (*n* = 10), based on their experiences of implementing FT. Interview data were transcribed verbatim, analysed using constructivist grounded theory, and informed by Fixsen's implementation science framework.

**Results:** Service providers highlighted a number of benefits for approximately two thirds of families across different diagnoses and mental health settings (AMHS/CAMHS/primary care). Sites varied in their capacity to embed FT, with key enablers identified as acquiring managerial and organisational support, building clinician skill, and establishing interagency collaboration. Implementation challenges included: recruitment difficulties, stresses in working with multiply-disadvantaged families, disruption in delivery due to the COVID-19 global pandemic, and sustainability concerns (e.g., perceived fit of FT with organisational remit/capacity, systemic and cultural barriers to change).

**Conclusion:** This study is only the second qualitative study ever conducted to explore practitioner experiences in implementing FT, and the first conducted within the context of an RCT and national research programme to introduce family-focused practise (FFP) for families living with PMI. The findings illuminate the successes and complexities of implementing FFP in a country without a “think family” infrastructure, whilst highlighting a number of important generalisable lessons for the implementation of FT, and other similar interventions, elsewhere.

## Introduction

Parental mental illness (PMI) is common, with 23% of all families having at least one parent who has, or had, a mental disorder (1), and a 41–77% lifetime risk for children of developing serious mental illness, physical illness, and impaired educational and occupational outcomes (2). Traditionally, both in Ireland and in other jurisdictions, these families have remained “invisible” and unsupported due to the segregation of adult and child mental health services (3, 4). Data on parenting status within mental health services is scarce (5), but early studies estimate that 25–68% of adult mental health service users are parents, and 35–60% of children presenting at child and adolescent mental health services have a parent with mental illness (6, 7).

Given the prevalence and burden of PMI–and in the context of the principles and values enshrined within the United Nations Convention on the Rights of the Child–there has been a growing recognition in many countries of the need to support families in order to protect children from developing mental disorders (8, 9). Reassuringly, a range of interventions has been developed (e.g.,targeting parents, children, whole family or peers), with evidence that they can decrease the risk of developing mental disorders for children by up to 40% and reduce referrals to child protection services (10, 11). Family Talk (FT), in particular, has been identified as a key intervention with promising evidence of effectiveness in improving parent and child understanding of, and communication about, mental illness and child internalising symptoms (9, 10, 12–14). FT is a whole-family, 7-session, manualised, clinician-facilitated, psycho-educational, and strengths-based approach designed to improve family communication and resilience (15), and has been implemented in recent years in several countries as part of national initiatives to support families where a parent has mental illness (e.g., the USA, Costa Rica, Colombia, the Netherlands, Greece, Scandinavia, Iceland, and Australia) (15).

Nevertheless, we know from the translation of other evidence-based psychosocial programmes that positive outcomes achieved in controlled research settings may not always be replicated within mainstream service settings (16, 17). According to Fixsen, the implementation of practise change typically involves a lengthy recursive process of six (non-linear) stages, including “exploration,” “installation,” “initial implementation,” “full implementation,” “innovation,” and “sustainability,” with each stage presenting its own unique set of challenges (18). Within the context of family-focused practise (FFP) for families with PMI—and including our own research–a number of implementation barriers have been noted, including: (1) the socio-political context (e.g., lack of policy/practise guidelines, dedicated funding); (2) organisational culture (e.g., siloed adult and child mental health services, ideological differences, under-resourced mental health teams); (3) clinician skill/attitudes (e.g., professional training typically based on a biomedical, crisis-oriented, individualised model of care); and (4) service user/families' willingness to participate (e.g., stigma, fear of losing custody, lack of awareness of impact of PMI on children) (5, 19–23). Research has found that implementation of FFP is erratic and unsystematic even within countries with established “think family” initiatives and legislation that mandates the identification and support of families with PMI (24–26). For instance, less than half of all clinicians in adult mental health services (AMHS) in Norway identified the parental status of service users despite acknowledging it to be a mandatory task (25), thereby indicating that changes in legislation or attitudes alone, do not necessarily lead to change in practise.

To date, only one published qualitative study of clinicians/managers' experiences in implementing FT has been conducted, despite FT being delivered as part of national initiatives in several countries (15). Eleven clinicians in Sweden were interviewed to explore their experiences of delivering FT to families living with parental psychosis. Several benefits were indicated, including increased family understanding of, and communication about PMI, and the utility of the FT manual in equipping clinicians to ask about patients' parenting capacity and children's well-being. Nevertheless, high rates of refusal and attrition were noted, and clinicians reported that some parents with psychosis lacked insight into the impact of their mental illness on their children. In addition, in a recent paper, the FT programme developer, William Beardslee, reported on his team's experience of delivering FT to parents with depression in the US and while this was not a qualitative paper involving interviews with clinicians, the importance of the clinicians' skill was highlighted, including their capacity to engage parents in the initial phase, build a partnership with families, and develop a shared, strengths-based, family narrative (27).

Whilst only one previous study has examined service-provider experiences of implementing FT, a small number of studies have reported on family experiences, which may help to inform workforce practise (28–32). Work by Pihkala et al. (30) and Strand et al. (32) showed that families (parents and children) have reported a number of benefits across a range of mental disorders, although there was some indication that those with BPD or low-functioning psychosis were more likely to struggle with establishing a therapeutic alliance and/or exhibit a lack of understanding/insight into the impact of their mental illness on their children. Parents indicated that factors enabling engagement included having a trusted and skilled professional to mediate family conversations, and timeliness, structure and flexibility of the intervention, while stigma and fear (e.g., being perceived as an incompetent parent) were often significant barriers to participation. However, it should be noted that all of these studies were conducted in psychiatric settings in Sweden, a country with legislation to support families with PMI and which has implemented FT as part of a national “think family” initiative since 2006 (29). In addition, small sample sizes, a limited range of informants, and an overall lack of cultural diversity, restricts the transferability of the findings and underscore the need for qualitative analyses to be undertaken across a wider variety of settings and contexts.

Ireland lags behind most European countries and also Australia, in its lack of legislation and/or a national “think family” policy/practise guidance to support families with PMI (24, 26, 33–36). Moreover, mental health provision in Ireland is severely underfunded when compared with European counterparts, with services operating at between two-thirds to three quarters of recommended staffing levels (37, 38). In the earlier phase of this research (2017–2018), we conducted a scoping study of FFP across adult (*n* = 114) and child (*n* = 69) mental health services in the Republic of Ireland and found that support for families was either non-existent, in the planning stages or *ad hoc* and small scale (4). In addition, the 2019 census for psychiatric units in Ireland provided statistics on 2,000+ inpatients (e.g., age, marital status, diagnosis, socioeconomic status), but failed to include their parental status (39), thereby highlighting a persistent lack of service awareness. Similarly, a recent qualitative study conducted with psychiatric nurses in Ireland (*n* = 14), identified several barriers to FFP, including lack of practise standards to identify service users as parents, no available structured approach, and an absence of appropriate training (3).

The funding provided by the national Health Service Executive (HSE) for the current “PRIMERA” research (**P**romoting **R**esearch and **I**nnovation in **M**ental h**E**alth se**R**vices for f**A**milies and children) was crucial in supporting the first endeavour to systematically implement FFP for families with PMI in Ireland. The aims of PRIMERA were to: (1) identify/develop, implement, and evaluate family-focused interventions for families with PMI; and (2) inform a “think family” care delivery agenda within mental health services in Ireland. Therefore, following an initial scoping and installation phase, we sought to introduce FFP into mental health provision in Ireland through the implementation and evaluation of FT (utilising a randomised controlled trial, qualitative and economic analyses) (4, 40). This qualitative study is one of two which were nested within a randomised controlled trial (RCT) of FT. The objective of this study was to identify and explore with clinicians and managers the barriers and facilitators to implementing and sustaining FT across adult, child (AMHS/CAMHS) primary care and child protection services in Ireland. A companion paper reports family experiences of FT across sites.

## Methods

This qualitative study of practitioner experiences of implementing FT was conducted in the context of an RCT of FT, and was analysed using constructivist Grounded Theory to identify and organise themes, and informed by Fixsen's implementation science framework and the Medical Research Council (MRC) guidance for complex interventions (18, 41, 42). Details of the RCT protocol and registration can be seen at the following link https://trialsjournal.biomedcentral.com/articles/10.1186/s13063-021-05199-4; (40).

### Participants and Settings

A purposive sample of mental health clinicians (*n* = 31), and managers (*n* = 10) were identified and recruited for participation in the study, based on their experiences of delivering FT to 55 families within the RCT.

The larger RCT included 86 families (139 parents, 221 children) in 15 sites across Ireland, involving AMHS, CAMHS, primary care psychology, and child protection/welfare services (40). Families were block randomised, on a 2:1 ratio, to the FT intervention (*n* = 56) or to a treatment as usual control group (*n* = 30), and assessed at baseline and 6-month follow up. At follow up, attrition was 37%, the rate of which doubled due to the impact of the COVID-19 lockdown restrictions (23 vs. 45%). Eligible families were those with a child aged 5–18 years and a parent with a formally diagnosed mental disorder. Eighty per cent of service-users were attending AMHS and 20% were receiving antidepressant medication or primary care psychological support under the care of a General Practitioner (40). Due to the high risk of intergenerational transmission of mental disorders (2), and a desire among stakeholders to increase family-focused collaboration between traditionally segregated adult (AMHS) and child mental health services (CAMHS) (4), we included families where children attended CAMHS or primary care services for mental health issues, as well as families where children were not involved with mental health services. Families were excluded if the parent/family was in a state of crisis/instability (e.g., hospitalised, active psychosis/addiction, contentious separation) (40). The 55 families included in service provider reports, had a similar profile to the larger RCT sample in terms of age, gender, mental disorder, and site/location (Table 1).

**Table 1:** Characteristics of families in RCT (*N* = 86).

	***N* (%)**
PMI gender (female)	73 (85)
PMI mean age (SD)	41.01 (7.09)
Lone parent	42 (49)
**Mental illness**	
– Anxiety/depression	55 (64)
– Bipolar	15 (18)
– BPD	9 (10)
– Psychosis	5 (6)
– PTSD	2 (2)
**Length of episode**	
– <6 months	16 (18)
– 6–12 months	15 (17)
– 1–2 years	11 (13)
– >2 years	44 (52)
Child gender (female)	120 (55)
Child mean age	10.27 (5.28)
**Child mental health**	
– CAMHS	42 (19)
– Other psychology/family service	50 (23)
– No service	127 (58)
Family social disadvantage^a^	65 (76)

*BPD, Borderline personality disorder; CAMHS, Child and adolescent mental health service; PMI, Parent with mental illness; PTSD, Post-traumatic stress disorder*.

a *Social disadvantage compared to Irish norms and calculated based on: income, employment, family size, lone parenthood, education and household ownership. In 2019, 17.8% of the population were defined as being socially disadvantaged (43)*.

**Figure 1:**
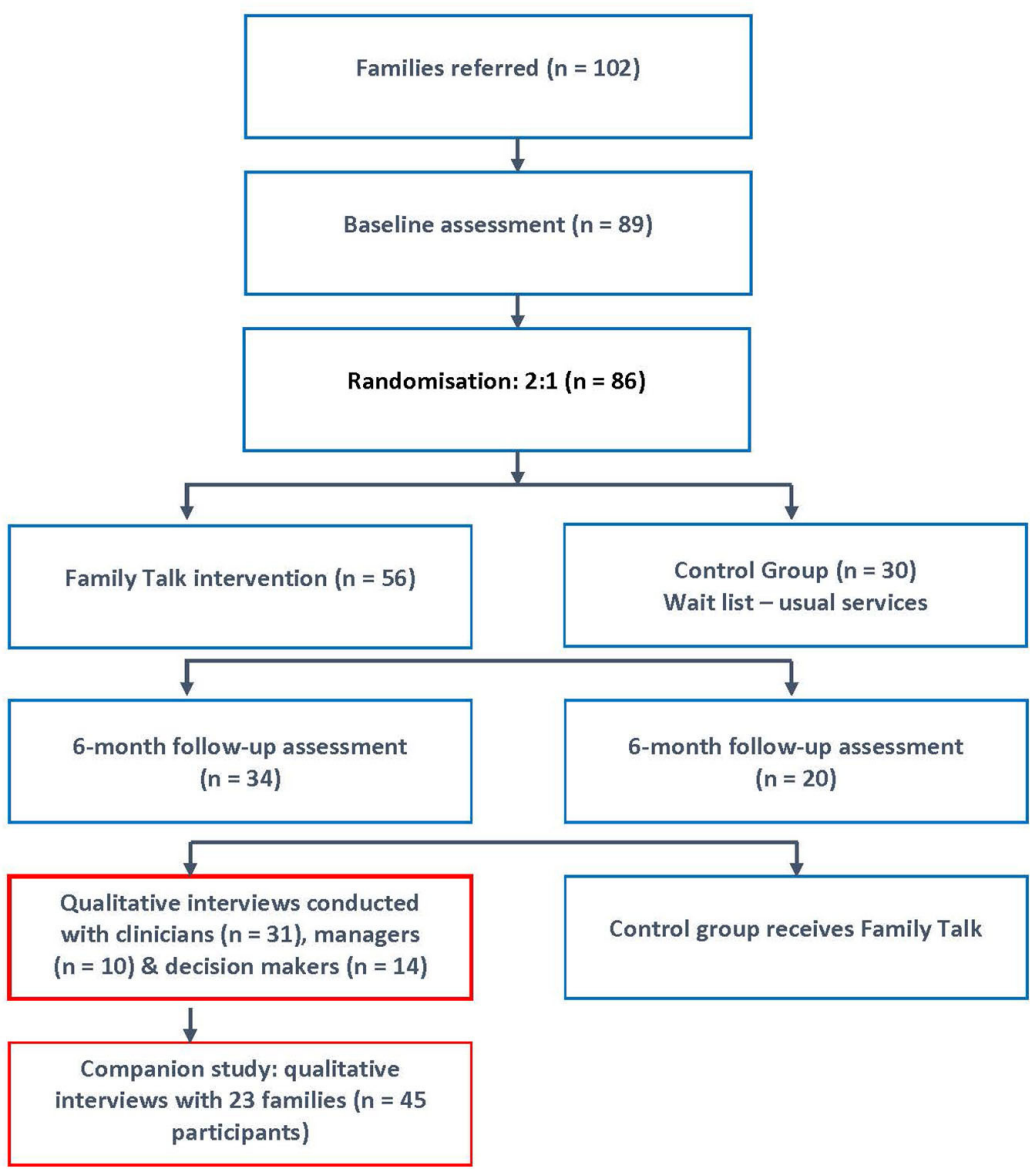
Study flow diagram from RCT to qualitative studies.

Participating sites were eligible to participate in the research if they had secured managerial support to implement FT, and had identified a lead person to coordinate clinicians, oversee training, plan family recruitment, organise regular peer supervision and be a point of contact with the research team. Clinicians delivering FT were required to have at least 3 years' experience in working within adult, child mental health and/or protection services; have completed the online training in FT (www.emergingminds.com.au), attend monthly supervision, and recruit families and/or facilitate FT. Families were recruited by clinicians in each site from their existing waiting lists. FT was delivered in an outpatient clinic and/or in the home by an FT clinician (40). Ethical approval (for both the RCT and qualitative study) was obtained from four research ethics committees including the research institution where the research was carried out [name withheld for anonymous peer review], the HSE, Tusla child protection agency and Saint John of God's Hospitaller Services. The flow of participants from recruitment through the RCT to the qualitative studies is shown in Figure 1.

Clinicians and managers were selected for interview based on service setting (e.g., AMHS, CAMHS, primary care, Tusla child protection agency), professional discipline (e.g., social work, psychology) and site location. All 15 sites were approached and interviews were secured with participants from five sites that recruited 10+ families each, from 3/5 sites that recruited ≤3 families, and from 1/5 sites that did not recruit any families. Three sites could not be contacted and three declined interview due to FT clinicians either leaving the service or having competing demands on their time due to the COVID-19 pandemic. Most of the 31 clinicians interviewed were female (*n* = 27), parents (*n* = 25), aged 31–50 years (*n* = 26), with 14 employed in AMHS, 14 in CAMHS and 3 in primary care and the Tusla child protection agency. More than three quarters were employed as social workers, three as social care workers, and the remaining five as clinical nurse specialists and psychologists. On average, they had been employed as practitioners for 15 years (SD = 6.7), with most (24/31) having worked in multiple settings (e.g., AMHS, CAMHS, and child protection services).

Ten managers were also interviewed, half of whom were female, six employed in AMHS, three in CAMHS and one in primary care psychology. Most (*n* = 6) were principal/senior social workers, two senior clinical psychologists, one systemic family therapist, and one general manager.

### Data Collection and Analysis

All participants provided written informed consent to participate in a one-to-one, semi-structured interview or focus group. Eight managers and eight clinicians participated in an individual interview while two managers and 23 clinicians were interviewed across five focus groups. The focus groups typically lasted ~1.5 h (with a break if so required), while one-to-one interviews with clinicians and managers lasted 30–45 min. Most interviews were conducted using online platforms (all managers, 24/31 of clinicians) due to the COVID-19 pandemic restrictions. An interview schedule/topic guide was devised to investigate: (1) stakeholders' experiences of facilitating/implementing FT; (2) key barriers and enablers to implementation; and (3) factors mediating the longer-term sustainability of FT/FFP in their service/in Ireland. The interviews were conducted by CM, who had met with all service providers several times previously during the exploration and installation phases of FT implementation (4). Interviews were audio recorded and transcribed verbatim.

The data were uploaded to MAXQDA software (44) and analysed using constructivist Grounded Theory in order to identify and organise themes (41). Analysis was informed by Fixsen's implementation science framework and the MRC guidance for complex interventions (18, 42). Data were analysed using line-by-line and focused coding, constant comparison of codes to find similarities and variations within categories and hierarchical linking of categories to generate super-ordinate (or overarching) themes. All of the interviews were read by CM and MF, CM coded and analysed all of the data, while three authors (MF, SMcGa, SOC) independently assessed the reliability of coding on 25% of the transcripts, with more than 90% inter-rater agreement. Disagreements were resolved by discussion. Trustworthiness of the analytic process was also enhanced by audiotaped interviews, verbatim transcription, audit trail of code generation, clear description of sampling procedures, participants and settings, theoretical saturation, and seeking disconfirming cases. Reporting adhered to COREQ guidelines (Consolidated Criteria for Reporting Qualitative Research) (45).

## Results

Three main themes and a number of subthemes therein, were identified, as outlined below (Table 2).

**Table 2:** Experiences of implementing Family Talk.

**Themes**	**Subthemes**
Facilitators to implementation	Organisational and managerial support Structured approach of FT Clinician skills and experience Seeing the benefits of the programme Role played by research/research team
Barriers to implementation	Engaging and retaining families – Family challenges – Clinician and organisational barriers – COVID-19 and research barriers – Variation across sites
	Delivery challenges
Sustainability of FT/FFP in Ireland	Site continuity plans FT fit with service remit and as part of FFP suite of supports Longer-term sustainability of FFP

*FFP, Family-focused practise; FT, Family talk*.

### Theme 1: Facilitators to Implementation

Clinicians indicated a number of factors as key to the successful implementation of FT including: organisational/managerial support; the structured approach of the intervention; clinician experience and skills; seeing the benefits of the work for families, clinicians and the wider service; and being part of a high profile and well-funded research programme.

#### Organisational and Managerial Support

Ten of the 15 sites recruited families for the RCT, with five sites recruiting 90% of all families (See Table 3). Sites that were more successful were more likely to be led by an FFP champion with strong networking and team-building skills, who had secured support from a Consultant Psychiatrist. In addition, FFP champions promoted interagency liaison amongst AMHS, CAMHS, Tusla, and primary care services which, in turn, facilitated recruitment, shared delivery, and learning. They also engaged in regular awareness-raising and buy-in efforts with management/colleagues to raise the profile of FT within their organisation through, for instance, promoting FT successes during multi-disciplinary team (MDT) meetings. They also established a referral structure for FT and held regular FT peer supervision meetings. Supervision was seen as important in increasing clinician competence and sharing storeys of successful outcomes for families helped to motivate clinicians in their recruitment and delivery efforts. Moreover, clinicians in these areas were given time to complete the training, engage in recruitment and FT facilitation, and attend supervision. It should be noted that sites that recruited more families were more likely to have joined the PRIMERA collaborative research programme in 2018, which gave them more time to train suitable clinicians and identify families, compared to other sites that only joined in mid/late 2019 and only a few months before the onset of the COVID-19 pandemic restrictions.

**Table 3:** Site characteristics.

**Site**	**Date joined study**	***N* family recruits**	**% family withdrawals**	**No. trained family talkers**	**Interagency effort**	**Service(s) involved**
1	Mid 2018	39	33	16	Yes	AMHS, CAMHS, PC, Tusla
2	Late 2018	15	19	5	No	AMHS
3	Mid 2018	14	12	18	Yes	CAMHS, AMHS, Tusla
4	Early 2019	13	17	10	Yes	CAMHS, AMHS, PC
5	Mid 2018	10	7	5	No	AMHS
6	Late 2019	2	2	1	No	AMHS
7	Late 2019	2	0	8	Yes	AMHS
8	Mid 2018	1	0	6	Yes	AMHS, recovery college
9	Mid 2019	2	5	1	No	Tusla
10	Late 2018	3	5	3	No	CAMHS
11	Mid 2018	0	–	3	Yes	AMHS, CAMHS
12	Late 2018	0	–	1	No	Tusla
13	Late 2018	0	–	2	No	Tusla
14	Late 2019	0	–	2	No	Tusla
15	Early 2019	0	–	3	No	AMHS

*AMHS, adult mental health services; CAMHS, child and adolescent mental health services; PC, primary care*.

“*Bringing them* [AMHS, CAMHS, Tusla and primary care] *all together for supervision every five weeks…discussing cases of dynamics and challenges. They also have the peer supervision and support…The work was seen as important.”* (Manager 5, AMHS, Site 1)

“*My consultant psychiatrists and my team are excellent–and she hears that this work is done. And she's delighted! But she's one of the few psychiatrists who I've seen think systemically.”* (Clinician 10, AMHS, Site1)

“*More recent referrals have come from team members…that probably has a lot to do with a few more of the talks from myself, a team meeting generating referrals…one of the consultants in the team was quite eager.”* (Clinician 3, CAMHS, Site 1)

#### Structured Approach of FT

All clinicians/managers appreciated the structured, yet flexible, approach that FT provided in working with families. They also valued its evidence base and its manualised, no-cost, online training. Most also highlighted the importance of the psycho-education provided, and indicated that the skills they had gained were transferrable, although some noted that additional face-to-face training might be helpful for managing more complex cases.

“*I thought the training was really good. I thought it was very accessible… I see the children and the parents get a lot from it… The checklist is really helpful…The structure is invaluable. It's really easy to evidence the work that I'm doing.”* (Clinician 8, AMHS, Site 2).

“*The fact that it was free, it was online, it's brief, that we could do it ourselves, it didn't require investment from the services–all those things appealed to us.”* (Manager 9, AMHS, Site 5)

“*I do think given the complexity of cases, you do need to modify, but the structure is there, and the structure is very accessible to most people. And that's one of the big strengths to it.”* (Clinician 14, AMHS, Site 1)

#### Clinician Skills and Experience

Clinicians with prior experience of working in both AMHS and CAMHS were more committed to FFP implementation, having observed at first hand the transgenerational effect on children when they became service users. In addition, cross agency experience gave clinicians confidence and competency in working with the whole family, and facilitated interagency collaboration and co-delivery of FT, which considerably enhanced family recruitment and the quality of programme provision. Furthermore, most participants were social workers and believed that their professional training equipped them to be more persistent with family work when compared with other disciplines on mental health teams; for instance, they felt more competent in assessing family readiness for FT; establishing a positive relationship with families before and during FT; and in working with multiple family members.

“*I spent time in both AMHS and CAMHS. You would see people being referred and you would see there was an inter-generational connection. What you often see is a history of parental mental illness and how that's impacted on them growing up.”* (Clinician 29, AMHS, Site 1)

“*What I liked about it was having the mum and dad and the others all in the room together because while this may be new for some clinicians, it's not odd for the family, because that's the way they work as their every day.”* (Manager 1, Primary Care, Site 4)

“*From my point of view, co-working works really well. The adult mental health practitioners being involved is really important because the children are very badly affected, so having this model of working on those cases, I'll be working with that going forward.”* (Clinician 11, Tusla, Site 1)

#### Seeing the Benefits of the Programme

##### Benefits to Families

An important and frequently reported implementation driver for clinicians/managers, was the benefits they had witnessed in approximately two thirds of the families with whom they were working; these included: reduced worry and stigma, a greater understanding of the impact of PMI on family members, a new family narrative around the parents' illness, and improved family communication. Clinicians indicated that parents/partners were typically surprised/upset by how much their children had been affected by tense/volatile home situations, and had hidden their worries and concerns to avoid burdening parents. For children, having their reality acknowledged, was significant as children were usually told nothing was wrong. As parents became more cognisant of their children's needs, family members were motivated to reduce levels of anger/arguments, and to relate to each other in more warm, caring and fun ways, thereby leading to reduced stress and increased family well-being. Clinicians further indicated that the improved family interactions/relationships assisted the PMI's personal and parental confidence and well-being.

“*I think parents being able for the first time to hear their kid's opinions, and that they have opinions on it, they do have questions, and they're not in the dark–that does have a positive impact. Parents can become upset. I have had parents who cry in the feedback session. They can't believe they* [children] *knew what was happening, but there is some motivating factor in that for recovery. One parent I was working with for over a year had not shown a massive shift, but whatever it was about hearing feedback from her kids, and questions about her mental health, it seemed to motivate her. It did make a difference to her recovery.”* (Clinician 4, AMHS, Site 3)

“*Their life is totally different. The mum had a lot of guilt and shame around her being a mental health patient. It was the first time she talked to the girls and they talked about the frustrations of mum not being available. She's able to speak to both the girls now. Mum is able to cook everyday when she couldn't before so life has become a lot more predictable, which is exactly what they wanted–so hugely beneficial for them.”* (Clinician 10, AMHS, Site 1)

“*For the kids themselves, just to be given that space to talk and have their own voice heard is huge… Because the kids know without maybe knowing what the words are for it, but they know that there's something going on in the household…Takes a huge weight off their shoulders…In one family, both girls were actually blaming themselves for mum's illness because their aunt had told them it was their fault that mum was having relapses.”* (Clinician 1, CAMHS, Site 3)

“*That was the best thing he [service user] had done he said and because of the communication with his family, he's doing quite well again. He's more aware of the need to communicate.”* (Clinician 15, AMHS, Site 1)

##### Benefits for Clinicians and the Wider Service

Most clinicians also believed that FT was beneficial for themselves and for their service. FT was reported to be enjoyable and rewarding and had helped to allay long-held ethical concerns about not addressing the needs of family members. In addition, several clinicians noted that FT worked well as a stepping stone for early identification of vulnerable families within their service, could be easily added to treatment plans, and was useful in signposting families to additional supports if required.

“*It's definitely a hugely beneficial piece of work… I could feel it as a practitioner, and they could feel it as a family.”* (Clinician 1, CAMHS, Site 3)

“*If you think about it, this intervention is almost social justice. We're doing what we believe is right in developing children's rights.”* (Clinician 10, AMHS, Site 1)

“*It was overall positive and really valuable work.”* (Clinician 3, CAMHS, Site 3)

#### The Role Played by the Research/Research Team

Clinicians and managers indicated further that a significant motivating factor for their involvement in FT training and delivery—and another key implementation driver—was the fact that the research was funded by the HSE (national health service in Ireland) and involved a multi-site, national programme aimed at addressing a major service gap in Ireland (i.e., developing FFP for families with PMI). Participants also clearly appreciated the wide range of advocacy and support activities undertaken by the research team to scaffold site buy-in, implementation, and family engagement. These included: co-developing a complementary online resource hub to assist clinicians in working with families; co-producing brochures and posters to recruit families; hosting/facilitating access to FFP workshops/masterclasses; co-delivering presentations to site managers and MDTs; providing regular updates by e-zines; and promoting the study through local and national media to raise public and service awareness on the topic (4). Thus, the early installation and implementation of FT was a joint collaboration between the research team and site stakeholders (4).

“*What attracted it to us was the fact that it was supported by research, it was multi-site, it was a broader ‘Think Family’ agenda which appealed to us… The sense of being part of something bigger. There was a support structure there and we wouldn't have done this in a systematic way unless we were part of the research study.”* (Manager 9, AMHS, Site 5)

“*More recent referrals have come from team members, and that has a lot to do with a few more of the talks by the research team coming into the service.”* (Clinician 3, CAMHS, Site 3)

“*It was great to be part of the research. I feel it was a very exciting time and you guys are doing such an incredible job…I definitely intend to keep going. I would absolutely love to see it more evolved in Ireland. I'm a big believer in it.”* (Clinician 4, AMHS, Site 3)

### Theme 2: Barriers to Implementation

#### Engaging and Retaining Families

Engaging and retaining families was the primary challenge faced by service providers, and was one which was exacerbated by the COVID-19 restrictions. Clinicians indicated that three to four families had to be approached for every one successfully recruited, and in ten sites there were three or less families recruited (Table 3). Overall, 16% (16/102) of referrals to the RCT were withdrawn before randomisation due to their unsuitability for FT (e.g., child protection issues, parent relapse, family crises). Of the 56 families allocated to the intervention group, 6 did not start FT and 5 disengaged after attending <3 sessions, with 53% attending all sessions [mean attendance was 4.4 sessions (Sd 1.2)]. Participants identified a range of barriers to engagement and retention covering multiple family, clinician, organisational, pandemic, research, and systemic/cultural levels.

#### Family Challenges

Clinicians indicated that for many parents–including those who agreed to attend FT–mental health stigma and concerns about involving their children, was a major concern and key barrier to implementation. Many parents disagreed about what should be discussed with their children, while concerns around social worker involvement with their children, also inhibited engagement. A small number of children in CAMHS also were anxious about discussing the issue with their parents. In addition, many of the cases on waitlists were complex (e.g., long-term service users, socially disadvantaged) which may also have affected engagement and retention. Thus, extensive preparatory work by clinicians was needed to allay all of these concerns and fears. Clinicians also reported that some families disengaged before FT commenced/completed due to family crises (e.g., threat of homelessness, job loss), relapse in mental health symptoms, having other priorities or finding it too emotionally painful to hear from their children about the impact of their illness on them.

“*As much as we're trying to reduce the stigma of mental illness… It's a massive thing still in Ireland. Especially I think for the parents. I definitely think more open communication is essential in families.”* (Manager 3, AMHS, Site 2)

“*I think it's probably about five or six families that said no. Their reason for saying no was, ‘don’t like social workers'… or fear that I'm going to start doing a parent assessment and that someone will be speaking to their child.”* (Clinician 15, AMHS, Site 1)

“*The family withdrew… Maybe it was the difficulty of having to talk to her mum about how she was feeling about their relationship. They disengaged with CAMHS… And then COVID hit and to be honest, the crisis of the last couple of months… so that has been it.”* (Clinician 21, CAMHS, Site 4)

#### Other Barriers

All sites experienced a number of organisational barriers that affected the engagement and retention of families, although some struggled more than others. Resistance to FFP from colleagues was reported as common due to: heavy workloads, staff shortages/high turnover, ideological differences (e.g., perceiving FFP to be outside their service remit), and feeling ill-equipped to undertake family work due to the individualised, crisis-oriented focus of their professional training. Other barriers included: slow referral processes; difficulties in identifying PMIs; needing to re-secure buy-in with new consultants who rotated on a 6-monthly basis; and colleagues being supportive in theory but not in practise as demonstrated, for example, by their unwillingness to train in FT or to refer families, a tendency to discharge suitable families without notice, and being risk adverse in balancing service-user confidentiality/data protection concerns with family needs.

“*We've had locum six-month positions who are very good psychiatrists, but then they're gone. And they don't have any weight when they're here for six months and they are very dismissible”* (Manager 8, CAMHS, Site 10)

“*Some will say that's not our job, it's a luxury, it's time consuming…Most other disciplines are trained just to work with an individual. So whereas we're going into the messy family life and that's a very frightening thing for services and they'll say to you, ‘oh GDPR’… It's very much a pushback, people aren't comfortable with it at all.”* (Manager 4, CAMHS, Site 3)

“*Health services are reactionary. They deal with crisis after crisis… Which shows how slow we have been to look at preventive intervention… The other reluctance around this is that if you start looking at the psychological and social aspects of mental health, that may potentially reveal the delusion of psychiatry and the medical model.”* (Manager 10, AMHS, Site 8)

In addition, there was evidence in some sites that insufficient effort may have been invested in recruiting families, which led to some not engaging with FT. For instance, it was reported that FT may have been poorly explained to families, or that parents had been informed by “cold calling” rather than through the building of a prior relationship with them. In addition, several clinicians indicated that negative past experiences of mental health/child protection services amongst some families, had led to their disengagement. Furthermore, some families dropped out following lengthy delays to FT delivery as a result of the COVID-19 pandemic restrictions in Ireland, and particularly in sites where mental health clinicians were redeployed to frontline COVID-19 duties (46). While involvement in the research promoted implementation and recruitment in some regards (as discussed earlier), being involved in a time-limited RCT also hindered recruitment to some degree. For instance, some families did not wish to be in the control group or to complete questionnaires. One site conducted FT with several families (*n* = 7) but not as part of the RCT and, despite support from the research team, struggled to communicate to families the value of taking part in the research.

“*The main challenge was recruitment. It's because they* [colleagues] *didn't explain it properly to the parent.”* (Clinician 31, CAMHS, Site 3)

“*We have been hugely affected by COVID… And after so much work put into it* [FT]. *That's been hugely challenging.”* (Manager 6, AMHS, Site 1)

“*We had a certain amount of time to complete it because of the* [research] *timelines so there's that added pressure to find families and get them seen. Once that is gone, it will be very good to see this as an integral part of AMHS. I really hope that happens.”* (Clinician 12, AMHS, Site 1)

#### Variation Across Sites

Ten sites recruited three or fewer families, only one of which (site 11) withdrew from the research; they did so because clinicians did not see FT as being a fit with the type of systemic family work which they wanted to undertake. The remaining nine sites were all characterised by limited resources (e.g., few FT clinicians with little dedicated time), ideological differences, lack of a champion or practical support from colleagues, and/or lack of organisational readiness to engage families due to joining the study later in its lifetime and especially with the onset of the COVID-19 restrictions. Furthermore, eight of the ten sites had little history of interagency work, which possibly impeded recruitment. Notably, those sites in which child protection services collaborated with AMHS and CAMHS were more successful in engaging families than those who attempted to deliver FT without such interagency support; the latter struggled with clinician buy-in and family recruitment. Child protection practitioners/service providers in Ireland are typically not trained in mental health, and without interagency support, they may have felt less equipped to undertake family-focused mental health practise. In addition, given their limited resources and crisis-oriented focus, they may not have considered families with PMI to meet their criteria/threshold of a child being at risk.

“*I felt a bit overwhelmed…I was the only one that took on the Family Talk intervention even though I spent a lot of time advocating for it… If I was rolling out Family Talk maybe in six- or seven-months' time, I think I would have had more space and the team would have gotten to know me better and trusted me with some of the families to see what social work can do. Within the team, the role of social work was a very basic view of the role of social work* [e.g., form filling and applying for benefits/services rather than engaging families in interventions].” (Clinician 26, AMHS, Site 7)

“*Mum has mental health problems, a lot of trauma from her background… The family would really benefit from it [FT]. But Tusla said, no, it doesn't meet our threshold as Dad's a protective parent.”* (Clinician 28, Tusla, Site 13)

### Delivery Challenges

A small number of clinicians indicated that the family meeting, in particular, was stressful, due to the emotional content being shared, and the requirement to support parents and children spanning a broad age range.

“*What I found difficult was the family meetings, you were sitting with mum, a 16-year-old, an 11-year-old and a six-year-old in the room. You speak differently…You're still getting the essence across, but you're not being as frank about certain issues, or you're making it more child friendly because a child is there.”* (Clinician 4, AMHS, Site 3)

Fidelity to FT protocols was also a challenge, with frequent delays/disruptions due to the COVID-19 restrictions. In a small number of cases, clinicians adapted FT using online platforms, which facilitated individual parent and older teen sessions, but was not considered suitable for younger children or family sessions, and therefore completion of FT was delayed. In addition, for families with more complex needs, one third of clinicians indicated that they provided additional parent, child and family sessions beyond the 7-session model, and referred families to further services (e.g., individual/relationship counselling, family supports). As FT was frequently the first (and perhaps only) time parents and children spoke about living with PMI, parents/partners were often angry/upset during initial sessions, while some service users needed time to adjust to not being the sole focus of care. Child meetings were also extended (if time permitted) when complex issues or concerns were raised.

“*Due to the pandemic, I was unable to recommence Family Talk. It was impossible to start the individual meetings again and it just didn't flow straight into the family meeting. Otherwise, I feel the Family Talk would have been very successful.”* (Clinician 7, AMHS, Site 2)

“*A couple of families had a lot of issues, and they needed time–one session with the kids wasn't going to be enough… And they needed follow-on supports that I was able to refer them to.”* (Clinician 11, CAMHS, Area 3)

### Theme 3: Sustainability of FT/FFP in Ireland

#### Site Continuity Plans

Despite the disruptive long-run impact of COVID-19 (e.g., increased waitlists), six sites have continued to deliver FT beyond the research programme, while the remaining areas hope to use its principles in practise, subject to resource limitations. The top five recruiting sites (Table 3) appear best placed to sustain FT as managers/clinicians have: (1) introduced practise guidelines for engaging families to FT as part of routine service provision (e.g., during initial patient assessments); (2) promoted FT using service-user feedback; (3) encouraged new staff/colleagues to train in FT; (4) continued to deliver FT to families; and (5) held regular FT peer supervision.

“*We have continued receiving referrals for Family Talk and are continuing to deliver it to families. I am delighted that staff want it to become embedded in practice and our peer supervision group has become an established forum.”* (Manager 6, AMHS, Site 1)

“*We still continue here in CAMHS. I still fly the Family Talk flag as much as I can.”* (Manager 4, CAMHS, Site 3)

“*We are going to continue using it in CAMHS. I think it's a very useful service. But definitely the challenge is the recruitment.”* (Clinician 21, CAMHS, Site 4)

“*I still use it. I use it in everyday work now.”* (Manager 10, AMHS, Site 8)

“*The intervention is really great so it's definitely something that we're going to continue to do with families. It should have been here a long time ago.”* (Clinician 29, AMHS, Site 5)

#### FT “Fit” With Service Remit and as Part of FFP Suite of Supports

A key sustainability issue concerned the perceived “fit” of FT with service remit; while many stakeholders expected AMHS to be the most natural fit for FT–and with CAMHS/Tusla perceived as being more proficient at family work–success in implementing FT appeared to be mediated more by local site resources, organisational culture and the availability of a strong champion. A small number of CAMHS clinicians within one site viewed FT as a mid-level intervention which was not sufficient for complex cases while CAMHS clinicians in other areas, working with equally complex cases, believed FT was appropriate. In addition, four AMHS and CAMHS clinicians believed that while FT principles would inform their future practise, the FT intervention would be better delivered at primary care level, given their lower threshold for access (i.e., mild to moderate mental health presentations). Conversely, a clinician working in primary care psychology indicated considerable recruitment challenges due to a lack of willingness among parents with moderate anxiety/depression to acknowledge the impact of their difficulties on their children. This participant indicated that recruitment should be easier in AMHS where patients generally have a more defined diagnosis. Therefore, while FT was successfully delivered in all types of service—AMHS, CAMHS, Primary Care, Tusla—thereby reflecting a “no wrong door” approach to service provision (25), all sites experienced considerable implementation challenges, and many participants felt that siloed service provision had undermined their capacity to properly support families. The child protection agency, Tusla, in particular, experienced the most implementation difficulties, most likely due to families' fear of social services, although interagency collaboration was shown in this study to support their involvement.

“*I expected CAMHS to be very family focused. I've been really surprised that it had, like adults, become very much focused on the child is the problem and you fix the child. And you don't look at anything else.”* (Manager 4, CAMHS, Site 3)

“*I think for us to use it to inform our practise but as a package, it probably would work better as a prevention piece on the primary care level.”* (Manager 7, AMHS, Site 2)

“*This is the problem when you fragment service, and they're not integrated. Tusla is a separate agency. Adult and CAMHS are very separate. This shouldn't be. Because children, come out of one family yet the family might be attending three or four different services, which is part of the problem.”* (Manager 6, AMHS, Site 1)

Given the complexity of some family cases, it was advised that sustainability of FFP in the RoI would be enhanced if FT was implemented as part of a suite of lower and higher intensity interventions. As indicated, clinicians felt the need to deliver extra sessions to several families, and frequently referred to follow-on services/supports, including individual and couple counselling, family and youth services, parent programmes, men's groups, and dialectical behaviour therapy. It should be noted that 76% of families in the RCT were socially disadvantaged and therefore presented with a high level of need.

“*Some families probably need longer intervention… And then when the parent can't overcome stigma or family members are resistant, maybe something lower key in just talking with the parent might help also. But FT has been great for the families that come to it.”* (Manager 1, Primary care, Site 4)

#### Longer-Term Sustainability of FFP in Ireland: Systemic Barriers and Roadmap

For longer-term sustainability of FFP in Ireland beyond a small number of committed sites, all participants indicated that FFP is unlikely to flourish within the current medical, individualised, siloed, under-funded, crisis-oriented model of mental health care in Ireland which was perceived as encouraging services to believe it is not their core business to support families with PMI. Other systemic barriers noted by decision makers include a lack of data and accountability of how HSE funding is spent, and initiatives typically being introduced in an *ad hoc* manner with little infrastructural support.

**Figure 2:**
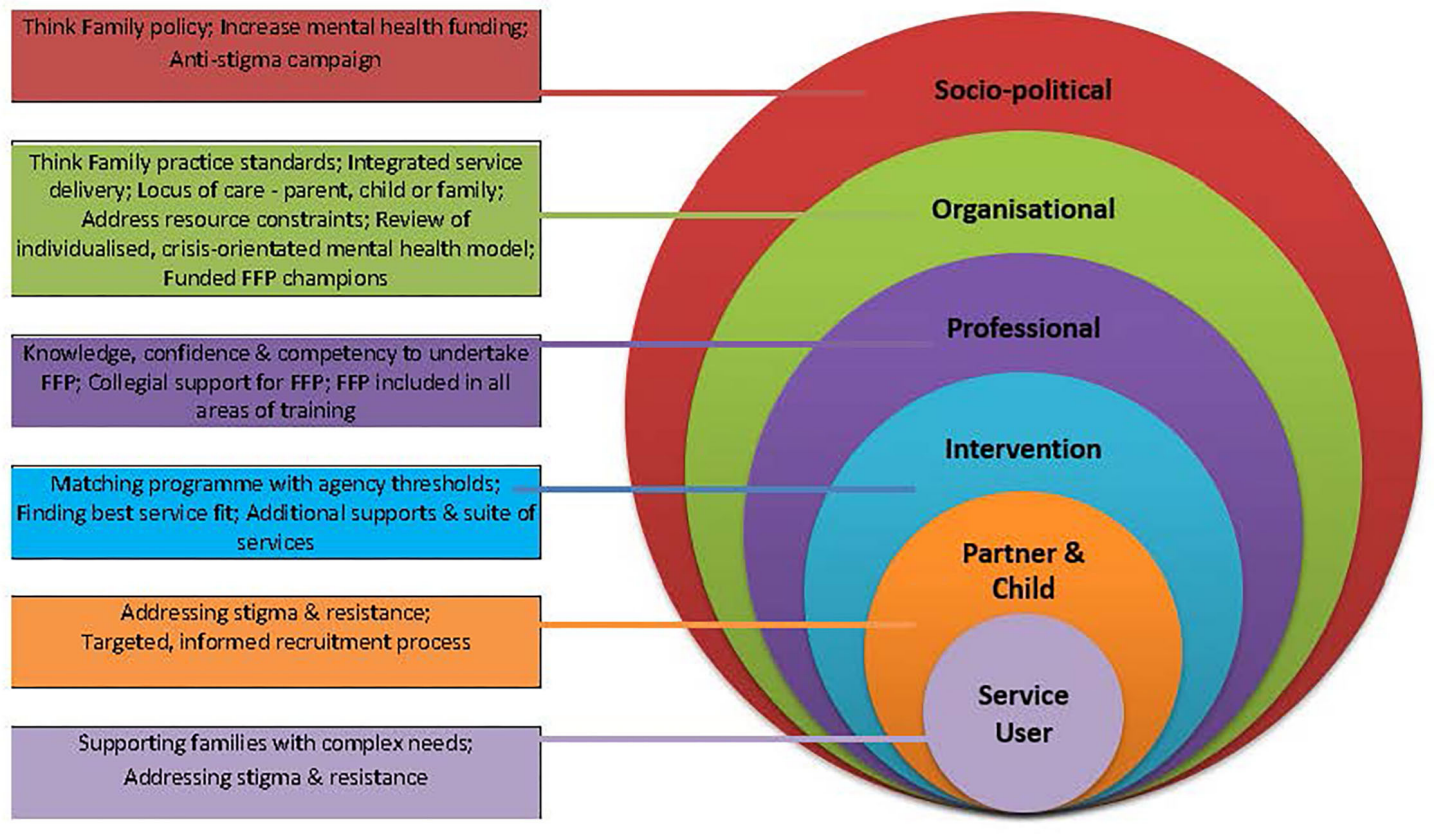
Multi-level approach to embed FFP.

All participants highlighted the need for a multi-level, public health approach to raise service and public awareness on PMI, including: introducing a national “think family” policy initiative/practise guidelines; providing dedicated funding for FFP, and mental health services more generally; launching media campaigns to reduce mental health stigma; addressing systemic/interagency barriers to change (e.g., including FFP within professional training across disciplines, auditing parenting status, and allowing time for FFP within, and across mental health services). In addition, change agents (champions) need access to senior management to effect change at frontline, operational, and strategic levels. Given the movement of personnel within the HSE, multiple FFP positions are needed to ensure sustainable practise (see Figure 2).

Interestingly, while all participants agreed that FFP was long overdue in the RoI, there was little consensus on the effectiveness of legislating/mandating FFP or the benefits of introducing standards which may, in practise, be reduced to a meaningless tick-box exercise with little benefits for families or clinical practise. Rather, participants emphasised the benefits of providing training in FFP, such as FT, and having managerial support to deliver FFP to families.

“*I think if you make this kind of thing mandatory or legislative, it adds a little bit to the scary factor, both for families and us working with them… I think a better investment is to train clinicians in it [FFP/FT] and then support them to do it, allow them time. But you need to move beyond the individual, medical model for that.”* (Manager 1, Primary Care, Site 4)

## Discussion

Service providers highlighted a number of benefits for the majority of families, while several key facilitators and barriers to implementation and sustainability were also identified. The benefits noted here corroborate those reported by a sample of family members (*n* = 45 from 23 families) who participated in a second qualitative study which is reported in a companion paper (ref withheld for purposes of anonymous review). The findings are also consistent with those of studies of clinicians and families who experienced delivering/attending FT in psychiatric settings in Sweden (30, 47). Perceived benefits for families in this study included: feeling heard and validated, reduced worry and stigma, a greater understanding of mental illness; improved parental confidence; and better family communication. Benefits were reported across different mental health settings (e.g., AMHS/CAMHS) and types of mental disorders and highlighted that FT was capable of being implemented in a country without a “think family” policy or dedicated FFP funding infrastructure.

Within the current study, clinicians/managers identified a number of facilitators and barriers to implementation, which build upon those identified in (the few) previous qualitative studies of FT delivery (28–32, 47), and which should help to inform the future implementation of FT/FFPs across countries. These might also usefully be tested as mediators/moderators within controlled trials. Five of the 15 sites recruited 90% of families (Table 3) and participants from these sites provided important insights into key facilitators. These included: the availability and drive of an FFP champion with managerial support; promoting interagency collaboration among AMHS, CAMHS, primary care, and child protection services in the area; engaging in regular awareness-raising and buy-in efforts with management/colleagues (e.g., FT on weekly MDT agenda and offered as part of care plan during initial assessments); encouraging clinicians to participate in FT training; setting up referral and supervision structures, and allowing clinicians sufficient time to engage in FT promotion, recruitment, and delivery activities. The use of multiple modes of recruitment (e.g., brochures, in-person invitations, phone-calls) also appeared to be linked to better family engagement. These findings are important in reinforcing the enablers of successful FFP implementation identified elsewhere, including building community capacity and interagency collaboration (5, 19), as well as targeting management, organisational policy, and professionals' attitudes, skills, and knowledge (5).

Another key facilitator to implementation was the structured, manualised approach of the intervention, and its freely available online training, which greatly increased its accessibility for busy professionals working across different geographical areas. Nevertheless, some clinicians indicated that they would have welcomed supplementary face-to-face training with international FT trainers, and would have liked the online training to show clinicians working with more complex cases (e.g., lone parenting/social disadvantage) and across a range of mental disorders. It should be noted that while clinicians in this study did not receive the 2-day, face-to-face FT training, they were required to undertake the online ‘Keeping Families and Children in Mind’ training to familiarise themselves in FFP prior to the FT training. They were also invited to several no-cost FFP masterclass/workshop events organised by the research team, whilst an online resource hub was also co-developed to supplement FT training in Ireland (e.g., providing resources on how to work with children, how to engage families) (4).

The level of clinician skill was another important enabler, including their capacity to engage parents in the initial phase, build a partnership with families, and develop a shared, strengths-based, family narrative (27). Most participants in this study linked their confidence and competency in FT delivery to their professional training in systemic approaches (e.g., social workers), and having previously worked within AMHS, CAMHS and child protection settings. Thus, the whole-family approach of FT dovetailed well with their attitudes and experience. Clinicians' self-efficacy beliefs have been indicated elsewhere as a key predictor of provider willingness to conduct FFP (48), and as such, addresses the “*not mine, not trained, too busy, too risky”* mindset that is a common barrier to FFP implementation (49). It is interesting to note that families in our companion study also highlighted the importance of clinician experience/competence and a non-judgemental and hopeful attitude, both of which were seen as helping to reduce stigma and promote family engagement.

A significant barrier to implementation across all sites related to difficulties in engaging families to take part in FT. Likewise, two other FT studies have also noted high refusal rates of up to 60% (13, 47). Clinicians indicated that barriers to engagement presented at family, clinician and organisational levels. Largely similar to FFP barriers noted elsewhere, family barriers included mental health stigma, parental fear and ambivalence about involving children, and families' complex presentations (i.e., PMI is only one of several presenting issues). Clinician/organisational barriers included limited resources/priority given to FFP; ideological differences; fragmented services; no champion to drive implementation; and/or little practical support from colleagues (3, 5, 24, 50). Interestingly, in our companion study of family experiences, several children reported that they were not informed about the purpose of FT and would have appreciated meeting the clinician before commencing sessions. Moreover, it appears that some families may have been approached before they were ready to engage (e.g., symptoms elevated, in denial/unaware of impact of their illness on children). These recruitment difficulties suggest that clinicians may further benefit from the development of FT/FFP training videos/protocols to promote effective engagement strategies and address potential barriers to participation and retention. For instance, addressing issues of stigma, readiness, consent, and confidentiality during the recruitment process and including quotes/videos from previous FT attendees, may help to improve engagement (50). In addition, a child-friendly recruitment approach that uses age-appropriate marketing literature and involved a meet-and-greet session with the facilitating clinician, might help to address children's concerns about attending. Similar protocols might also be usefully developed to promote organisational/clinician commitment to FT/FFP implementation, including, for instance, putting FFP on the weekly agenda and in careplans, discussing ideological concerns (e.g., confidentiality, data protection, service remit), gaining collegial support, and securing dedicated time to undertake FFP. It is important to note that several sites were gaining momentum in recruitment just as the COVID-19 emergency was starting and, for the same reason, those sites which joined the study at a later date, did not have an opportunity to engage families as they had intended (Table 3).

Clinicians identified some pressure points when delivering FT. A small number found that facilitating the family sessions was particularly intense (and occasionally volatile) given the range of perspectives and developmental stages of family members. Therefore, it might be useful if the online FT training provided advice on how to tailor the discussion when children of different ages (e.g., 6 vs. 16 year-old) are present. Secondly, most families indicated that they would have liked more child, family and follow-up sessions whereas one third of clinicians indicated that, for complex cases, they had already provided additional sessions beyond the 7-session model and had referred families to further services. The families' perspective most likely reflects their level of need (e.g., 76% were socially disadvantaged), as well as the general unavailability of mental health/family supports in the community, whereas the clinicians' perspective reflects working within a context of limited service resources for FFP. Most previous studies of FT have not mentioned the need for additional sessions or follow-on supports, but this may be due to their participants being largely middle class and relatively high functioning (12–14, 51, 52). There is evidence from two qualitative studies of FT that some parents with low-functioning psychosis and Borderline Personality Disorder may struggle to understand the impact of their illness on their children, and may require additional supports (31, 47). These supports may include extra psycho-educational sessions and/or complementary groups for patients and children, in order to share experiences and learn about their mental illness and its impact on their children (47).

The sustainability of FFP in Ireland was a recurring concern for all participants. Reassuringly, six sites have continued delivering FT beyond the research programme and have established structures to enhance its sustainability, such as integrating FT into organisational procedures and care plans, and providing continued supervision and training of new FT personnel. Therefore, these sites have moved beyond Fixsen's stage of initial implementation, and particularly Site 1, but they have not yet reached full implementation as sustainability is still vulnerable to champions leaving the service (18). The remaining eight sites indicated that they will either: (1) use the FT principles in practise (e.g., “think family” when working with a service user) but not deliver the whole intervention; or (2) deliver FT as individual clinicians, but without receiving much practical support from management/colleagues. Therefore, all sites indicated that implementation of FT has enhanced a “think family” mindset but there is significant variation in terms of embeddedness (18).

These sustainability concerns raise questions about the perceived fit of FT with organisational remit and capacity. In many ways, given the individualised model of care in AMHS/CAMHS, it was a significant paradigm shift for these services to deliver a whole-family intervention, such as FT. While service providers appreciated the benefits gained from the whole-family model, there were nevertheless indications that FT should be implemented as part of a flexible suite of lower and higher intensity interventions, as recommended by international experts in the field (53). Higher intensity interventions may be more suitable for families presenting with complex needs, while lower intensity interventions may appeal to organisations with limited resources/individualised model of care and/or where families have less need or parents are unwilling to involve their children in services. In some jurisdictions, the two-session, parent-only, “Let's talk about the children” (LT) intervention has been implemented in AMHS settings and has been shown to increase understanding of PMI (13). Nevertheless, in our companion study of family experiences, we found that FT allowed children (and partners) to reveal burdens and concerns that would likely have remained concealed with an intervention that only involved interacting with the service-user parent. Furthermore, two head-to-head RCTs of LT and FT found the latter to be more effective in reducing child emotional symptoms and improving the parent–child relationship (12, 13). Therefore, further dialogue is required on whether mental health services should adapt their remit to become less individualised, and more family-focused, and/or whether only lower intensity interventions should be implemented so as to fit in with current service constraints.

Another key sustainability issue is identifying the type of service that is best placed to deliver FT/FFP. While AMHS may appear the most natural fit (given that parents have a diagnosis], our results demonstrate that CAMHS, primary care, and child protection services can effectively deliver FT, thereby reflecting a “no wrong door” approach to FFP provision. Mediating factors in the current study were less related to type of service than to the availability of a champion and local site resources as well as organisational culture, and interagency collaboration. In Australia, where a range of FFP supports have been established for over 20 years, AMHS and primary care are the most common provider settings (24), but in general, there is a consensus that FFP is the responsibility of all services, whether adult- or child-focused (54).

## Strengths and Limitations

This study is just the second qualitative analysis of practitioner experiences of implementing FT, and the first conducted within the context of an RCT and national programme to introduce FFP for families with PMI across AMHS, CAMHS, primary care and child protection settings (in Ireland). A large and diverse sample of stakeholders (*n* = 41) was interviewed including clinicians and managers across a number of sites, including those that struggled with recruitment. The findings identified a number of barriers and facilitators to implementation and mirror the family experiences of FT reported here in our companion paper.

Limitations include the generalisability of the findings across different cultural contexts and settings. Unlike other jurisdictions where FT was longer established and/or there was prior legislation/FFP practise standards, FT was implemented in Ireland as a catalyst for a paradigm change in mental health provision for families with PMI. In addition, most sites involved AMHS or CAMHS staff so caution is advised, therefore, in generalising to other mental health/family support settings. Furthermore, most of the clinicians/managers were social workers and 80% had previous experience in working within AMHS, CAMHS and/or child protection settings, thereby potentially limiting generalisability to other disciplines and those without cross-agency experience. Importantly, there was some evidence that FT implementation (e.g., site buy in) had taken place because it was the focus of a national research programme funded by the HSE in Ireland. While some clinicians indicated that the RCT timeline also impeded recruitment, all RCTs are time-limited which means that some families were not ready to participate within the timeframe of the study or they did not wish to be part of the control group. Lastly, this is the first study of FT to be undertaken, in part, during a global pandemic. The COVID-19 lockdown restrictions halted recruitment, and seriously affected programme delivery and fidelity which led, in turn, to some family disengagement from services. Service providers were also interviewed during the height of the pandemic restrictions, which may have affected their perspectives given the impact of the pandemic on mental health in the general population (55, 56).

## Recommendations for Practise, Policy and Research

Benefits were reported for approximately two thirds of families across different diagnoses and mental health settings (AMHS/CAMHS/primary care/child protection), thereby reflecting a “no wrong door” approach to identifying and supporting families. Key implementation facilitators included: acquiring managerial and organisational support through awareness-raising and buy-in activities; building clinician skill in systemic practise; establishing interagency collaboration across AMHS, CAMHS and primary care; setting up referral and supervision structures, and allowing clinicians sufficient time to engage in FT promotion, recruitment and delivery activities. Recruitment difficulties may be targeted by addressing issues of stigma, readiness, consent and confidentiality during the initial engagement process with families and including quotes/videos from previous FT attendees (50). In addition, children's concerns about attending FT may be allayed by using age-appropriate marketing literature and setting up an initial meet-and-greet session with the facilitating clinician before the FT sessions begin. Organisational/clinician commitment to FT/FFP implementation may be enhanced by: putting FFP on the weekly agenda and in careplans, discussing ideological concerns (e.g., confidentiality, data protection, service remit), and securing dedicated time to undertake FFP. In some cases, it may be necessary to signpost families presenting with multiple disadvantage to additional supports following FT. Lastly, where it is difficult to secure organisational support to undertake family work such as FT, it is still important for practitioners to refer relevant families (parents and children) to online resources such as Emerging Minds^1^ and to family supports/services in the community.

The longer-term sustainability of FFP in Ireland, and elsewhere, requires a multi-level public-health response to address enduring political, cultural, organisational, and family barriers to change. Such a response would include: “think family” policy/practise standards; dedicated funding for FFP; managerial support to implement FFP; initiatives to reduce mental health stigma and recruitment barriers; and a continuum of FFP to broaden its capacity to identify families (Figure 2). “Think Family” policy/practise standards include: mandatory auditing of the parenting status of adult mental health users, balancing the priority given to patient confidentiality with unmet family needs, increased collaboration between traditionally segregated AMHS and CAMHS services, and equipping clinicians with time and resources to undertake FFP (5, 33).

Although FT has been implemented in many countries, this is only the second qualitative analysis of practitioner experiences in implementing the programme. Therefore, further qualitative research of practitioner (and family) experiences is required across different cultural/policy contexts, disciplines and settings. Further research is also needed to identify measures and/or supports that might increase family engagement, including, for example, developing and evaluating training videos that teach recruitment strategies. In addition, the facilitators and barriers to implementation identified in this study (and other qualitative analyses) could be tested as moderator/mediator variables in quantitative research.

## Conclusion

In order to develop FT, and more broadly FFP, beyond a small number of committed sites, its longer-term sustainability in Ireland (and elsewhere) requires a careful assessment of the perceived fit of interventions with organisational remit and capacity, and the development of a multi-level public-health response to address enduring political, cultural, organisational, and family barriers to change (Figure 2). While little is known to date about which specific factors are most effective in promoting FFP, it is likely that change across all levels is required as legislation/standards, or FFP training on their own, are not sufficient (18, 25, 57).

## Data Availability Statement

The original contributions presented in the study are included in the article/supplementary material, further inquiries can be directed to the corresponding author/s.

## Ethics Statement

The studies involving human participants were reviewed and approved by four Ethics Committees: the Social Research Ethics Committee in Maynooth University, Ireland (Reference Number SRESC-2018-100), the HSE Research Ethics Committee, Tusla Ethics Review Committee, and the Saint John of God's Research Ethics Committee. Written informed consent to participate in this study was provided by the participants' legal guardian/next of kin.

## Author Contributions

CM, MF, and SMcG conceived and designed the study. CM conducted interviews and coded transcripts, with 25% of transcripts independently coded by MF, SMcGa, and SO'C. CM prepared the initial draft, with subsequent drafts undertaken by MF, with input from CM, SMcG, and SMcGa. All authors contributed to the article and approved the submitted version.

## Funding

The PRIMERA research programme was funded by the Health Service Executive and the Maynooth University Higher Education Authority COVID-19 Costed Extension Fund.

## Conflict of Interest

The authors declare that the research was conducted in the absence of any commercial or financial relationships that could be construed as a potential conflict of interest.

## Publisher's Note

All claims expressed in this article are solely those of the authors and do not necessarily represent those of their affiliated organizations, or those of the publisher, the editors and the reviewers. Any product that may be evaluated in this article, or claim that may be made by its manufacturer, is not guaranteed or endorsed by the publisher.

## Footnotes

^1^Emerging Minds website–https://emergingminds.com.au/.

## Abbreviations

AMHS: adult mental health services
BPD: Borderline Personality Disorder
CAMHS: child and adolescent mental health services
FFP: family-focused practice/programmes
FT: Family Talk
HSE: Health Service Executive
LT: Let's Talk about the Children
MDT: multi-disciplinary team
PC: Primary Care
PMI: parents with mental illness
PRIMERA: **P**romoting **R**esearch and **I**nnovation in **M**ental h**E**alth se**R**vices for f**A**milies and children
PTSD: Post-traumatic stress disorder
RCT: randomised controlled trial
RoI: Republic of Ireland.
